# Gut microbiota of Brazilian *Melipona* stingless bees: Dominant members and their localization in different gut regions

**DOI:** 10.1371/journal.pone.0326546

**Published:** 2026-05-07

**Authors:** Amanda Tristao Santini, Alan Emanuel Silva Cerqueira, Nancy A. Moran, Helder Canto Resende, Weyder Cristiano Santana, Sergio Oliveira de Paula, Cynthia Canedo da Silva

**Affiliations:** 1 Department of Microbiology, Federal University of Viçosa, Viçosa, MG, Brazil; 2 Department of Integrative Biology, The University of Texas at Austin, Austin, Texas, United States of America; 3 Institute of Biological and Health Science, Federal University of Viçosa, Florestal, MG, Brazil; 4 Department of Entomology, Federal University of Viçosa, Viçosa, MG, Brazil; 5 Department of General Biology, Federal University of Viçosa, Viçosa, MG, Brazil; Universidad San Francisco de Quito - Campus Cumbaya: Universidad San Francisco de Quito, ECUADOR

## Abstract

The gut microbiome of eusocial corbiculate bees, which include honeybees, bumblebees, and stingless bees, consists of anciently associated, host-specific bacteria that play crucial role in nutrition, pathogen defense and host fitness. While the core microbiota of honeybees and bumblebees is well characterized, the composition, spatial organization, and evolutionary dynamics of the microbiota of stingless bees remain poorly understood. This gap is particularly evident in the diverse genus *Melipona*, where *Snodgrassella* and *Gilliamella*, ubiquitous symbionts of honeybees and bumblebees, appear rare or absent, indicating a shift in microbiota composition in these stingless bees. Here, we address this gap by characterizing the microbiota of multiple *Melipona* species using 16S rRNA amplicon sequencing of newly collected and previously published data from field-collected samples. We also mapped the spatial localization of the dominant microbiota members within the gut regions of *Melipona quadrifasciata anthidioides* through targeted dissection. The *Melipona* microbiota is dominated by members of the genera *Bifidobacterium*, *Lactobacillus*, *Apilactobacillus*, *Floricoccus*, and *Bombella*, with striking regional structure. *Apilactobacillus* and *Bombella* dominate in the crop, whereas *Apilactobacillus* and other members of the Lactobacillaceae are most abundant in the ventriculus. The ileum lacks *Snodgrassella* and *Gilliamella* but contains a putative new symbiont closely related to *Floricoccus*, as well as strains of *Bifidobacterium*, Lactobacillaceae (including *Apilactobacillus*), and *Bombella*. The rectum is dominated by *Bifidobacterium* and *Lactobacillus*. These findings reveal a distinct microbiota architecture in *Melipona* that differs from other corniculate bees yet retains compartment-specific specialization, suggesting an alternative symbiotic strategy that may reflect unique dietary ecology and evolutionary history. Understanding these patterns advances our knowledge of host-microbe symbiosis and provides a baseline for microbiome conservation in declining stingless bee populations.

## Introduction

The association between insects and microorganisms is vital for the diversification and evolutionary success of insects [1]. Among them, social bees host a diverse and specialized gut microbiota that is both host-specific and relatively conserved across species, as well as environmental bacteria [2]. The establishment of the gut microbiome results from a combination of social transmission among colony members and acquisition from environmental sources such as nesting materials and diet [2–4].

This gut microbial community contributes to multiple aspects of host biology, including nutrition (e.g., fermentation of pollen and nectar by lactic acid bacteria), immune modulation (recruitment or priming of host defenses against pathogens), pathogen defense (by competing exclusion or direct antagonism), and overall colony health and resilience [5,6].

Eusocial corbiculate bees comprise three clades: the honeybees (genus *Apis*), bumblebees (genus *Bombus*), and stingless bees (tribe Meliponini) [7]. Their gut microbiomes contain anciently associated, host-specific bacteria that can contribute to bee health [2,8,9]. In honeybees and bumblebees, gut communities are typically dominated by the symbionts *Snodgrassella* and *Gilliamella* in the ileum, which form biofilms on the gut wall and work in metabolic cooperation (e.g., *Gilliamella* breaks down complex sugars, *Snodgrassella* oxidizes fermentation products) [8,9]. In contrast, stingless bees, including the large Neotropical genus *Melipona*, frequently lack or have minimal occurrences of *Snodgrassella* and *Gilliamella* [4,9–14]. In *Melipona,* the functional roles of *Snodgrassella* and *Gilliamella* have been speculated to be replaced by previously unreported taxa [10], including a member of the family Streptococcaceae, close to *Floricoccus* and consistently found in *Melipona* species [10,13,14]. This shift in the microbiota composition may have functional consequences for host health, nutrition and ecology, yet the identity, abundance, spatial localization and functional roles of the dominant microbes in *Melipona* guts remain poorly characterized.

Here, we address this knowledge gap by integrating newly generated and published 16S rRNA amplicon sequencing datasets on gut bacterial communities of field-collected of several Brazilian stingless bees’ species. In addition, we determined the spatial localization of the dominant bacterial taxa across gut regions of *Melipona quadrifasciata anthidioides* Lepeletier, 1836*.* Our results add to the understanding of the shifts in microbiota structure that have occurred in *Melipona*, including a possible replacement of *Snodgrassella* and *Gilliamella* by previously unreported taxa.

## Materials and methods

### Sample collection

The sample collection was authorized by the Brazilian Environment Ministry (SISBIO/ICMBIO authorization number 87892−1). To infer the dominant members of *Melipona* microbiome, we collected bees from ten (10) populations (i.e., bees from the same species living at the same sampling location) across different locations in Brazil. The populations consisted of two *Melipona* species identified by comparison with known specimens and/or taxonomic keys [15] and five morphotypes whose identification was not confirmed (referred to as “*Melipona* cf. = *conferatum*”). The number of colonies sampled per population varied based on availability in each location, as shown in Supplementary S1 Table.

Each colony consisted of a beekeeping box, from which forager bees were collected from the entrance and placed in sterile tubes containing 95% ethanol. Five bees from each colony were surface-sterilized in 1% (v/v) sodium hypochlorite for 3 minutes, rinsed three times in sterile distilled water, and dissected under a stereomicroscope using sterile forceps. The dissected guts from the five individuals were then combined to form a single pooled sample. As a control, three *Apis mellifera* samples were processed and sequenced in parallel, as the gut microbiota of this species is well characterized in the literature. These controls showed no evidence of reagent contaminants and were used exclusively to verify sample integrity.

To assess the microbial diversity in each gut region we selected the *M. quadrifasciata* species the most studied *Melipona* species to date [6,12,13,15], highly available in our university. We collected forager bees from 3 different colonies in Viçosa – MG, Brazil (Supplementary S1 Table), and dissected the gut of ten bees into four regions: crop, ventriculus, ileum, and rectum. Each region was treated as a separate sample, resulting in a total of 40 samples (one rectum sample was later discarded).

### DNA extraction and sequencing

For all samples in this study, the total DNA was extracted using the NucleoSpin soil kit (Macherey-Nagel), preceded by a proteinase K treatment for 2 hours at 56 ºC, as described in previous work [10]. After extraction, the DNA was submitted for 250 bp paired-end amplicon sequencing at Novogene Corporation Inc (Sacramento, CA, USA) using an Illumina NovaSeq 6000 System. The primer pair 341F (CCTAYGGGRBGCASCAG) and 806R (GGACTACNNGGGTATCTAAT) was used to target the 16S rRNA V3-V4 regions.

### Bioinformatics and phylogenetic analysis

The newly generated sequencing data (SRA accession #PRJNA1076254) were processed together with previously published data (SRA accession #PRJNA678404) [10] using the DADA2 package (version 1.28) [16] in R 4.3.1, following the pipeline available at https://benjjneb.github.io/dada2/tutorial.html. The taxonomy was assigned to ASVs using a trained SILVA database (version 138.1 from November 2020) for bacteria. Sequences assigned to chloroplast, mitochondria, or eukaryotes were removed prior to analysis.

Furthermore, the most abundant and *core-like* ASVs (i.e., ASVs present in all *Melipona* populations analyzed) were submitted to BLASTN similarity searches against GenBank at NCBI Reference Sequence Database at which we could identify and download sequences from isolates aligned to them. Downloaded sequences were aligned using MAFFT version 7 [17], and the Maximum Likelihood phylogenetic tree was made with a bootstrap of 1,000 replications using IQ-TREE 2 [18]. This approach allowed us to infer the potential origins and phylogenetic placement of dominant ASVs in *Melipona* (S4 Table, S4 Fig).

### Statistical and community analysis

For data analysis, we used the R package “mctoolsr” version 0.1.1.9 (available at https://github.com/leffj/mctoolsr), “vegan” version 2.6–4 [19], and “ggplot2” version 3.4.2 [20]. The dataset was rarefied to 43,110 reads per sample, corresponding to the lowest sequencing depth among all samples, to ensure comparability across samples and minimize biases associated with uneven sequencing depth. Because this threshold matched the minimum sequencing depth, no samples were excluded, and the rarefaction level avoided over-rarefaction. Alpha diversity metrics (Shannon and richness) and beta-diversity analyses were calculated after rarefaction. Differences in alpha diversity among gut regions were assessed using Kruskal-Wallis rank sum tests, followed, when appropriate, by Dunn’s post hoc tests with Benjamini-Hochberg correction for multiple comparisons, as implemented in the dunn.test package (version 1.3.6) in R [21].

Beta-diversity patterns across gut regions were evaluated using Non-metric Multidimensional Scaling (NMDS) and Permutational Multivariate Analysis of Variance (PERMANOVA). NMDS ordinations were generated using the calc_ordination function in the mctoolsr (version 0.1.1.9) package, based on Bray-Curtis dissimilarities. PERMANOVA was performed with the adonis2 function in the vegan package (version 2.6–4), using Bray-Curtis distances and 1,000 permutations to test for differences in community composition.

## Results

### Overall microbiota composition

**Fig 1:**
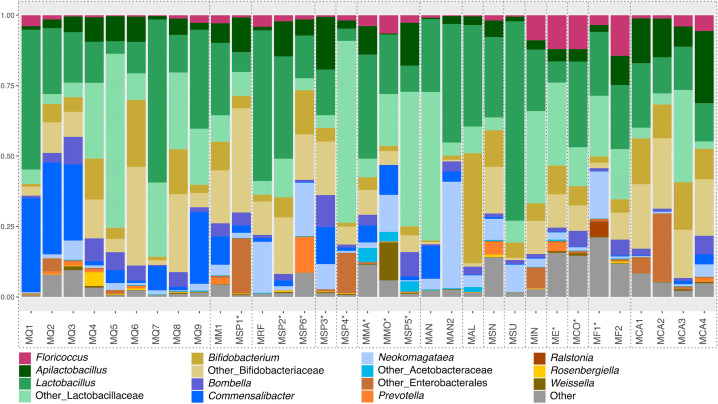
Mean relative abundance of gut bacterial genera in *Melipona* populations classified using SILVA database. Each column represents the mean relative abundance of each population (represented in S2 Fig). ‘Other_Lactobacillaceae’ refers to bacteria assigned to Lactobacillaceae that could not be identified at the genus level. Similarly, ‘Other_Acetobacteraceae’ refers to bacteria assigned to Acetobacteraceae that could not be identified at the genus level. ‘Other_Enterobacterales’ refers to bacteria only identified at the order level. ‘Other’ are bacteria in lower abundance. See S1 Table for population and collection information. Populations grouped by dotted lines are considered from the same *Melipona* species. *Species whose identification was not confirmed.

The microbiota of Brazilian *Melipona* bees is more similar within the same subgenera and biome (S1 Fig), consistently comprising Acetobacteraceae, Bifidobacteriaceae, Lactobacillaceae, and Streptococcaceae (S2 Fig). Across all bee populations analyzed, a recurrent set of bacterial genera was detected, including *Apilactobacillus*, *Bifidobacterium*, *Bombella, Commensalibacter*, *Floricoccus*, *Lactobacillus*, and *Neokomagataea*. A few samples contain other environmental genera, such as *Prevotella*, *Rosenbergiella*, and *Weissella* (Fig 1, S3 Fig).

### Diversity and community structure

For *Melipona quadrifasciata*, alpha diversity differed among gut regions. Kruskal-Wallis tests on the Shannon index indicated significant differences among gut compartments (χ² = 14.9, df = 3, *p* = 0.0019; S4 Fig and S5 Table), with the ileum showing the highest values. In contrast, richness did not differ significantly among gut parts (Kruskal-Wallis χ² = 5.06, df = 3, *p* = 0.17; S4 Fig and S6 Table), and post hoc Dunn tests confirmed the absence of significant pairwise differences (*p* > 0.05 for all comparisons).

**Fig 2:**
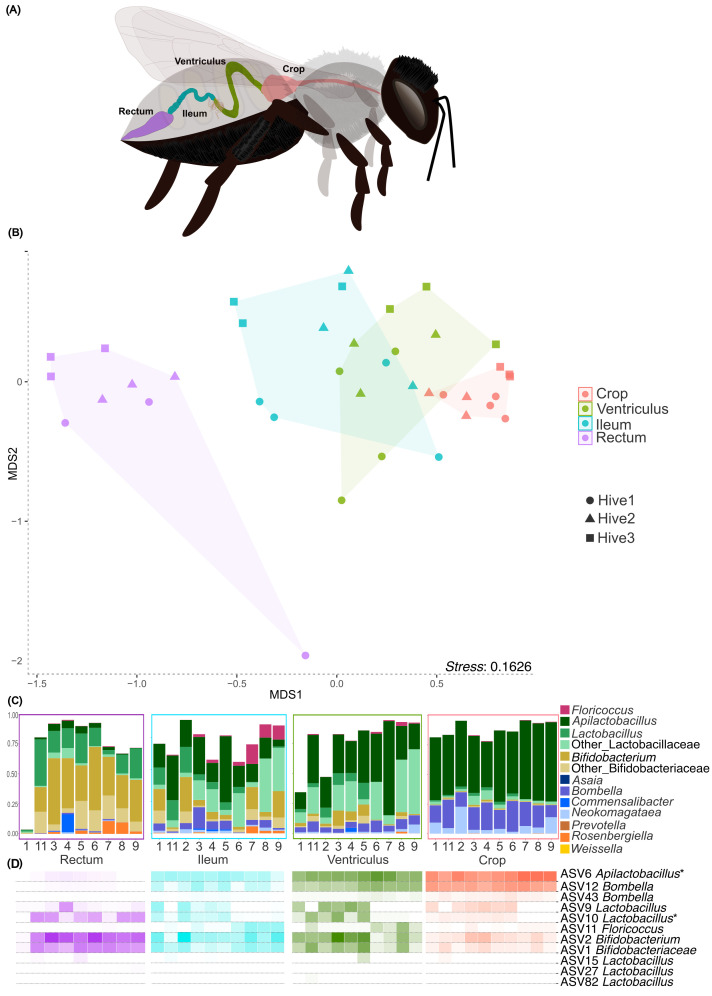
Microbial community of gut regions of *M. quadrifasciata anthidioides.* **(A)** Schematic figure of *Melipona quadrifasciata* gut. **(B)** NMDS based on ASV relative abundance (Bray-Curtis dissimilarity) in gut regions of bees from three colonies. **(C)** Relative abundance of dominant bacterial genera classified using SILVA database, in each gut region. **(D)** Heatmap of *Melipona core-like* ASVs in each gut region classified using SILVA database. *^1^ASV6 was classified as *Apilactobacillus* using SILVA database but formed a clade with *Nicoliella* using Genbank Nucleotide Database sequences (see S4 Fig). *^2^ASV11 was classified as *Floricoccus* using SILVA database but formed a clade with yet undescribed Streptococacceae isolates close to *Floricoccus* using Genbank Nucleotide Database sequences (see S4 Fig). All illustrations elements were created by the authors using Inkscape (version 1.3.2)..

Beta-diversity analysis revealed clear differences in the bacterial community composition along the gut axis (Fig 2A). The NMDS based on the Bray-Curtis dissimilarity matrix separated samples primarily by gut region rather than by source colony (Fig 2B; NMDS stress = 0.1626), and PERMANOVA analysis revealed significant differences among gut regions (F = 5.25, R^2^ = 0.31, df = 3, *p* < 0.05), with all pairwise comparisons being significant except between ventriculus and ileum (S2 Table). These results indicate that, although overall alpha diversity is comparable in terms of richness, gut regions harbor significantly different bacterial community compositions in terms of beta diversity.

### Gut region specific diferences in *Melipona quadrifasciata*

The genera that are most abundant in *Melipona* together account for more than 70% of the community within individual gut regions, but their relative contributions vary strongly along the gut axis (Fig 2C). In *M. quadrifasciata*, the crop is dominated by *Apilactobacillus* (55.7%), *Bombella* (18.4%), and *Neokomagataeae* (7.36%) (Fig 2B); the ventriculus by *Apilactobacillus* (35%), other Lactobacillaceae (22.4%), *Bombella* (6.3%)*,* and Bifidobacteriaceae (6%); the ileum by Lactobacillaceae (44.6% in total, including *Apilactobacillus* and *Lactobacillus*), Bifidobacteriaceae (15%, including *Bifidobacterium*), *Bombella* (5.3%)*,* and *Floricoccus* (5%); and the rectum by Bifidobacteriaceae (46%, including *Bifidobacterium*) and Lactobacillaceae (23.2%, including *Apilactobacillus* and *Lactobacillus*). These quantitative patterns are consistent with the significant PERMANOVA results and highlight strong gut region–specific differences in the structure of the bacterial communities.

Along the gut axis, a sequential decrease is observed in the relative abundance of *Apilactobacillus* (S3 Table) from the crop to the rectum, and *Bombella* is also more abundant in the crop compared to ventriculus and ileum. In contrast, *Bifidobacterium* and other Bifidobacteriaceae show the opposite pattern, increasing in relative abundance from the ventriculus to the rectum, where they are the main colonizers along with *Lactobacillus*.

### Core-like microbiota

Of the total 1,690 ASVs detected in the samples, 11 ASVs were present in all *Melipona* species analyzed (100% prevalence across species and sampling locations) and are therefore considered putative core members (hereafter referred to as *core-like* microbiota ASVs) (Fig 2D). These 11 ASVs are related to *Bifidobacterium*, *Bombella*, *Floricoccus, Lactobacillus*, and *Apilactobacillus*. Among them, ASV6 (*Apilactobacillus*) and ASV12 (*Bombella*) are the most prevalent in both crop and ventriculus; ASV9 and AS10 (*Lactobacillus*) are more abundant in the ventriculus, ileum and rectum, while ASV11 (*Floricoccus*) is more prominent in the ileum; and ASV1 and ASV2 (Bifidobacteriaceae and *Bifidobacterium*, respectively) show increased relative abundance in the ileum and rectum. Although the other *core-like* ASVs have lower abundances in each gut region, they are consistently present in all analyzed regions of *M. quadrifasciata*.

**Fig 3:**
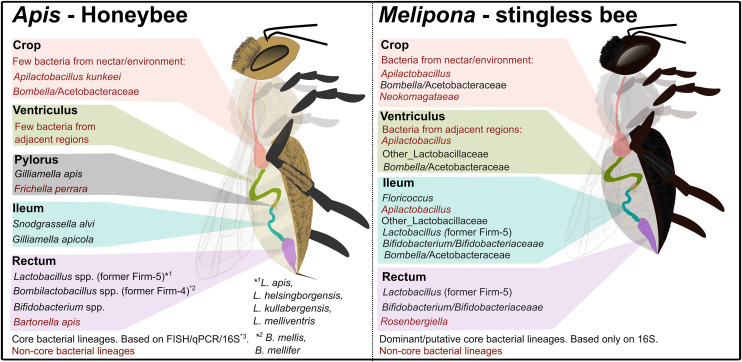
Comparative schematic of gut microbiota composition in *Apis* and Brazilian *Melipona* bees across different gut regions [2,3,22,23]. All illustrations elements were created by the authors using Inkscape (version 1.3.2).

Overall, Brazilian *Melipona* bees lack core bacterial lineages typically associated with honeybees, including *Gilliamella*, *Snodgrassella* and *Bombilactobacillus* (former Firm-4). Instead, they have acquired new putative *core-like* bacterial lineages, such as *Floricoccus* (Fig 3).

### Phylogenetic relationships of dominant ASVs

We constructed phylogenetic trees for the dominant and most abundant *Melipona* ASVs to distinguish bacteria consistently associated with bees from those typically found in other environments (S5 Fig). ASVs of the *Lactobacillus*, *Bombella* and *Bifidobacterium* groups in *Melipona* are related to those found in other bees, including isolates from bumblebees [24]. The *Floricoccus* ASV, although close to environmental isolates, formed a distinct clade together with strains previously isolated from *Melipona* [14]. Similarly, the *Apilactobacillus* ASVs are closely related to *Nicoliella spurrieriana*, a bacterium isolated from *Tetragonula carbonaria*, an Australian stingless bee [25]. These observations point towards two putative new clades associated with stingless bees (Fig 2C, S5 Fig).

## Discussion

The microbiota of *Melipona* differs from that of other eusocial bees, with rare or no occurrence of the canonical gut symbionts *Snodgrassella* and *Gilliamella*, corroborating previous observations [4,10,12–14,26]. The Brazilian *Melipona* microbiota is dominated by *Bifidobacterium*, *Lactobacillus*, *Apilactobacillus*, *Floricoccus,* and *Bombella*, which are consistently present in all bee populations analyzed. This study provides the first comprehensive analysis of the *Melipona* gut regions and their microbial composition. We specifically chose to analyze *M. quadrifasciata* due to its widespread occurrence in Brazil, and its role in honey production and agricultural pollination. In addition, the abundance of research available on this species [6,12,13,15] enabled us to assess the consistency between the microbial communities across the gut regions and the dominant members of the *M. quadrifasciata* microbiome. In the anterior gut, the crop (the sugar-rich honey stomach of bees) and ventriculus are enriched in fructophilic and nectar-associated taxa [25,27]. The crop microbiota is dominated by *Apilactobacillus* and *Bombella*, whereas the ventriculus is dominated by *Apilactobacillus* and other Lactobacillaceae, as well as *Bombella*. These microorganisms are typical lactic acid and acetic acid bacteria associated with sugar-rich substrates and the hive environment [4,28]. Their high prevalence in the crop and ventriculus suggests key roles in the processing of nectar and honey, including sugar fermentation, acidification, and possibly protection against osmotolerant yeasts or spoilage microbes [24,25,27–29]. In line with previous work showing that the anterior gut of bees often harbors a mixture of environmental and transient bacteria [30], our results indicate that *Melipona* have recruited fructophilic lineages that are well adapted to the highly osmotic and carbohydrate-rich conditions of the crop and ventriculus.

In other social bees, over 90% of the gut microbiota is found in the hindgut, consisting of ileum and rectum [22], and is dominated by *Snodgrassella*, *Gilliamella*, and specific Lactobacillaceae and Bifidobacteriaceae lineages [22,31]. In *M. quadrifasciata*, the rectum is dominated by *Bifidobacterium* and *Lactobacillus*, consistent with a conserved role of these taxa in the fermentation of complex carbohydrates, production of organic acids, and colonization resistance in the hindgut [3,31]. By contrast, the ileum in *M. quadrifasciata* departs from the honeybee and bumblebee patterns: instead of *Snodgrassella-Gilliamella*, it contains a community dominated by Lactobacillaceae, Bifidobacteriaceae, *Bombella* and a putative new symbiont closely related to *Floricoccus* that has already been isolated from *Melipona* [14]. In honeybees, *Bombella* and *Apilactobacillus* are largely confined to the crop [24,32,33], whereas in *Melipona* they extend into the ileum, suggesting that these lineages may have expanded their niche and partially replaced the metabolic and defensive roles typically fulfilled by *Snograssella* and *Gilliamella* in other corbiculate bees. This pattern is consistent with comparative studies in Afrotropical and Neotropical stingless bees showing repeated losses and gains of canonical gut phylotypes and their replacement by alternative lineages shaped by host phylogeny, nesting environment, and foraging ecology [4,12].

Together, these results support a scenario in which *Melipona* have undergone an evolutionary shift in their hindgut symbionts, which rather than retaining the *Snodgrassella-Gilliamella* biofilm that dominates the ileum of honeybees and bumblebees and contributes to energy metabolism, organic-acid turnover and pathogen defense [3,22], *Melipona* appear to rely on alternative Lactobacillaceae, Bifidobacteriaceae, *Bombella* and *Floricoccus* lineages to perform analogous functions. The drivers of this shift are not yet fully understood, but may include differences in nesting substrates (e.g., resin-rich cavities and cerumen pots), long-term storage of honey and pollen, diet breadth, and colony structure, all of which have been implicated as modulators of stingless bee microbiota composition [4,34].

While our study provides a detailed view of gut microbiota composition across *Melipona* species and gut regions, our findings should be viewed as a first step toward understanding gut microbiota organization in those bees, and several aspects warrant further investigation. Our sampling covered multiple *Melipona* species and populations across Brazil, but additional populations and regions would help to assess how generalizable these patterns are across the full distribution of the genus. Because we focused on forager bees, our results primarily reflect the microbiota associated with this worker caste; as reported for honeybees, gut community composition can vary with caste, age, and task, and microbiota profiles in in-hive workers or queens may differ from those described here [2,22]. In addition, we used 16S rRNA amplicon sequencing of pooled gut samples from forager bees, which is well suited for characterizing overall community structure but does not resolve strain-level diversity or directly reveal functional gene content. Finally, our hypothesis about the functional roles of the dominant bacterial taxa is based on their phylogenetic relatedness to characterized bacteria in other bees and environments. Future studies incorporating broader geographic sampling, metagenomic and metabolomic approaches, and experimental colonization assays will be valuable to refine and test these functional inferences.

## Supporting information

S1 Table Information of collection, species name and source of the *Melipona* samples analyzed in the present work. (PDF)

S2 Table PERMANOVA based on the Bray-Curtis dissimilarity matrix comparing the differences in the microbial community composition between the gut regions of *M. quadrifasciata anthidioides.* (PDF)

S3 Table Mean relative abundances and corresponding percentages of the main bacterial genera detected in each gut region of *Melipona quadrifasciata.* (PDF)

S4 Table GenBank sequences used for analysis. (PDF)

S5 Table Results of Kruskal–Wallis and Dunn’s post hoc tests comparing alpha diversity (Shannon index) across gut regions of *Melipona quadrifasciata.* (PDF)

S6 Table Results of Kruskal–Wallis and Dunn’s post hoc tests comparing alpha diversity (richness) across gut regions of *Melipona quadrifasciata.* (PDF)

S1 Fig NMDS plot based on ASV relative abundance using a Bray-Curtis dissimilarity matrix, illustrating bacterial community composition across different *Melipona* species and biomes. (PDF)

S2 Fig Most abundant families in *Melipona* spp. gut microbiota. Each sample represents a pool of 5 bees per box per site of study.
(PDF)

S3 Fig Most abundant genera in *Melipona* spp. gut microbiota. Each sample represents a pool of 5 bees per box per site of study.
(PDF)

S4 Fig Bacterial alpha diversity of the gut regions of *M. quadrifasciata.* (PDF)

S5 Fig Phylogenetic trees of the most abundant ASVs (including the 11 core ASVs) found in *Melipona* bee populations. (PDF)
