# Supplementary material for: Gut microbiota of Brazilian Melipona stingless bees: Dominant members and their localization in different gut regions
Source: PLoS One. 2026 May 7;21(5):e0326546. doi: 10.1371/journal.pone.0326546 (PMC13152157; doi:10.1371/journal.pone.0326546)
Supplement: S1 Table — (PDF) [file pone.0326546.s001.pdf]

S1 Table. Information of collection, species name and source of the *Melipona* samples analyzed in the present work.

Population Code	Number of Colonies	Sampling Biome	Sampling City	Sampling State	Bee Species	Sub-genus	Source*
ME	2	Amazon Forest	Rio Branco	Acre	*Melipona cf. eburnea*	*Michmelia*	New
MCO	4	Amazon Forest	Oriximiná	Pará	*Melipona cf. compressipes*	*Melikerria*	New
MCA1	3	Atlantic Forest	Castelo	Espírito Santo	*Melipona capixaba*	*Michmelia*	New[2*]
MCA2	3	Atlantic Forest	Domingos Martins	Espírito Santo	*Melipona capixaba*	*Michmelia*	New[1*]
MCA3	3	Atlantic Forest	Castelo	Espírito Santo	*Melipona capixaba*	*Michmelia*	New[2*]
MCA4	3	Atlantic Forest	Domingos Martins	Espírito Santo	*Melipona capixaba*	*Michmelia*	New[1*]
MF1	2	Cerrado	Goiânia	Goias	*Melipona cf. fasciculata*	*Michmelia*	New
MF2	2	Amazon Forest	Rio Branco	Acre	*Melipona fasciculata*	*Michmelia*	New
MMA	3	Atlantic Forest	São José	Santa Caratina	*Melipona cf. marginata*	*Eomelipona*	New
MMO	3	Atlantic Forest	São José	Santa Caratina	*Melipona cf. obscuriror*	*Eomelipona*	New
-	3	Atlantic Forest	Viçosa	Minas Gerais	*Melipona quadrifasciata*	*Melipona*	New[3]
MAL	3	Caatinga	Petrolina	Pernambuco	*Melipona asilvai*	*Eomelipona*	[12]
MAN	3	Atlantic Forest	Santa Tereza	Espírito Santo	*Melipona mandacaia*	*Melipona*	[12]
MAN2	3	Caatinga	Petrolina	Pernambuco	*Melipona asilvai*	*Melipona*	[12]
MIN	3	Amazon Forest	Iranduba	Amazonas	*Melipona interrupta*	*Melikerria*	[12]
MM1	3	Atlantic Forest	Viçosa	Minas Gerais	*Melipona mondury*	*Michmelia*	[12]
MQ1	3	Atlantic Forest	Magé	Rio de Janeiro	*Melipona quadrifasciata*	*Melipona*	[12]
MQ2	3	Atlantic Forest	São Sebastião do Paraíso	Minas Gerais	*Melipona quadrifasciata*	*Melipona*	[12]
MQ3	3	Atlantic Forest	Antônio dos Santos/Caeté	Minas Gerais	*Melipona quadrifasciata*	*Melipona*	[12]
MQ4	3	Atlantic Forest	Viçosa	Minas Gerais	*Melipona quadrifasciata*	*Melipona*	[12]
MQ5	3	Atlantic Forest	Cotia	São Paulo	*Melipona quadrifasciata*	*Melipona*	[12]
MQ6	3	Atlantic Forest	São Paulo	São Paulo	*Melipona quadrifasciata*	*Melipona*	[12]
MQ7	3	Atlantic Forest	Santa Tereza	Espírito Santo	*Melipona quadrifasciata*	*Melipona*	[12]
MQ8	3	Atlantic Forest	Cotia	São Paulo	*Melipona quadrifasciata*	*Melipona*	[12]
MQ9	3	Cerrado	Passos	Minas Gerais	*Melipona quadrifasciata*	*Melipona*	[12]
MRF	3	Atlantic Forest	Santa Tereza	Espírito Santo	*Melipona rufiventris*	*Michmelia*	[12]
MSN	3	Amazon Forest	Iranduba	Amazonas	*Melipona seminigra merrilae*	*Michmelia*	[12]
MSP2	3	Atlantic Forest	São Paulo	São Paulo	*Melipona cf. rufiventris*	*Michmelia*	[12]
MSP1	3	Atlantic Forest	Cotia	São Paulo	*Melipona cf. mondury*	*Michmelia*	[12]
MSP3	3	Atlantic Forest	Cotia	São Paulo	*Melipona cf. scutellaris*	*Michmelia*	[12]
MSP4	3	Atlantic Forest	São Paulo	São Paulo	*Melipona cf. bicolor*	*Eomelipona*	[12]
MSP5	3	Atlantic Forest	Cotia	São Paulo	*Melipona cf. marginata*	*Eomelipona*	[12]
MSP6	3	Amazon Forest	Iranduba	Amazonas	*Melipona cf. rufiventris*	*Michmelia*	[12]
MSU	3	Caatinga	Mossoró	Rio Grande do Norte	*Melipona subnitida*	*Melipona*	[12]

[*]All bee samples collected for this work are listed as “New”. [1*]Bees from the same population collected in different seasons (summer/winter). [2*]Bees from the same population collected in different seasons (summer/winter). [3]Bees collected for the study of the gut sections. These bees are from the same populations as the MQ4 [12].
