# Supplementary material for: Gut microbiota of Brazilian Melipona stingless bees: Dominant members and their localization in different gut regions
Source: PLoS One. 2026 May 7;21(5):e0326546. doi: 10.1371/journal.pone.0326546 (PMC13152157; doi:10.1371/journal.pone.0326546)
Supplement: S2 Table — (PDF) [file pone.0326546.s002.pdf]

S2 Table. PERMANOVA based on the Bray-Curtis dissimilarity matrix comparing the differences in the microbial community composition between the gut regions of *M. quadrifasciata anthidioides*.

Source	Df	Sum of Squares	R2	F	Pr (>F)
Crop x Ventriculus	1	0.34977	0.16274	3.49880	0.00199
Crop x Ileum	1	0.61699	0.22428	5.20436	0.00199
Crop x Rectum	1	1.47043	0.38016	10.42658	0.00099
Ventriculus x Ileum	1	0.18936	0.07356	1.42922	0.13486
Ventriculus x Rectum	1	1.04267	0.28248	6.69284	0.00099
Ileum x Rectum	1	0.68807	0.18743	3.92140	0.00099
All gut parts	3	2.15244	0.31038	5.25089	0.00099
