# Supplementary material for: Gut microbiota of Brazilian Melipona stingless bees: Dominant members and their localization in different gut regions
Source: PLoS One. 2026 May 7;21(5):e0326546. doi: 10.1371/journal.pone.0326546 (PMC13152157; doi:10.1371/journal.pone.0326546)
Supplement: S3 Table — (PDF) [file pone.0326546.s003.pdf]

S3 Table. Mean relative abundances and corresponding percentages of the main bacterial genera detected in each gut region of *Melipona quadrifasciata*.

Gut part	Mean (relative abundance)	Mean percentage	Genus_clean
crop	0.00	0.25	*Floricoccus*
crop	0.56	55.70	*Apilactobacillus*
crop	0.01	1.50	*Lactobacillus*
crop	0.03	3.26	Other Lactobacillaceae
crop	0.01	0.81	*Bifidobacterium*
crop	0.00	0.50	Other Bifidobacteriaceae
crop	0.18	18.45	*Bombella*
crop	0.00	0.01	*Commensalibacter*
crop	0.07	7.37	*Neokomagataea*
ventriculus	0.01	0.83	*Floricoccus*
ventriculus	0.35	35.16	*Apilactobacillus*
ventriculus	0.05	4.55	*Lactobacillus*
ventriculus	0.22	22.40	Other Lactobacillaceae
ventriculus	0.04	4.13	*Bifidobacterium*
ventriculus	0.02	2.05	Other Bifidobacteriaceae
ventriculus	0.06	6.33	*Bombella*
ventriculus	0.00	0.34	*Commensalibacter*
ventriculus	0.02	1.85	*Neokomagataea*
ileum	0.05	4.59	*Floricoccus*
ileum	0.22	22.27	*Apilactobacillus*
ileum	0.07	7.24	*Lactobacillus*
ileum	0.15	15.46	Other Lactobacillaceae
ileum	0.10	10.31	*Bifidobacterium*
ileum	0.05	4.61	Other Bifidobacteriaceae
ileum	0.05	5.28	*Bombella*
ileum	0.00	0.37	*Commensalibacter*
ileum	0.01	1.49	*Neokomagataea*
rectum	0.00	0.27	*Floricoccus*
rectum	0.03	3.10	*Apilactobacillus*
rectum	0.18	17.67	*Lactobacillus*
rectum	0.02	2.48	Other Lactobacillaceae
rectum	0.33	32.83	*Bifidobacterium*
rectum	0.12	11.91	Other Bifidobacteriaceae
rectum	0.01	0.55	*Bombella*
rectum	0.02	1.77	*Commensalibacter*
rectum	0.00	0.28	*Neokomagataea*
