# Supplementary material for: Gut microbiota of Brazilian Melipona stingless bees: Dominant members and their localization in different gut regions
Source: PLoS One. 2026 May 7;21(5):e0326546. doi: 10.1371/journal.pone.0326546 (PMC13152157; doi:10.1371/journal.pone.0326546)
Supplement: S4 Table — (PDF) [file pone.0326546.s004.pdf]

S4 Table. GenBank sequences used for analysis.

Specie name	Accession	Isolate substrate
	Apilactobacillus tree	
Nicoliella spurrieriana	NR_184611.1	*Tetragonula carbonaria* gut
Nicoliella sp.	OR978307.1	*Lavandula angustifolia* flowers in Spain
Apilactobacillus xinyiensis	NR_181939.1	Honeybee
Apilactobacillus ozensis	NR_113194.1	Mountain flower
Apilactobacillus apisilvae	NR_184610.1	*Austroplebeia australis* whole bee homogenate
Apilactobacillus quenuiae	NR_179014.1	Bee gut (*Augochlorella pomoniella*)
Apilactobacillus timberlakei	NR_179013.1	Flowers of *Abutilon* species
Apilactobacillus bombintestini	NR_174266.1	*Bombus ignitus* gut
Apilactobacillus apinorum	NR_126247.1	*Apis mellifera* honey stomach
Apilactobacillus nanyangensis	NR_179357.1	*Apis mellifera* gut
Apilactobacillus kunkeei	NR_026404.1	Fermented grape juice
Apilactobacillus zhangqiuensis	NR_179374.1	*Apis mellifera* gut
Fructilactobacillus fructivorans	NR_113640.1	Type strain of *Fructilactobacillus fructivorans*
	Lactobacillus tree	
Lactobacillus acetotolerans	NR_117073.1	Chicken crop
Lactobacillus sp. strain Mbhsr5	HM53808.1	Honeybee stomach
Lactobacillus sp. strain MbHmro5	HM534807.1	Honeybee stomach
Lactobacillus sp. strain ESL0731	CP113921.1	*Scaptotrigona polysticta* gut
Lactobacillus sp. strain ESL0700	CP113930.1	*Scaptotrigona polysticta* gut
Lactobacillus sp. strain ESL0677	CP113946.1	*Melipona interrupta* gut
Lactobacillus sp. strain ESL0680	CP113945.1	*Melipona seminigra* gut
Lactobacillus sp. strain MbHmro1	HM534806.1	Honeybee stomach
Lactobacillus sp. strain ESL0785	CP113916.1	*Melipona lateralis* gut
Lactobacillus sp. strain Alhm2to11	HM534787.1	Honeybee stomach
Lactobacillus bombicola	NR_136436.1	Bumblebee gut
Lactobacillus apis	NR_125702.1	Honeybee gut
Lactobacillus panisapium	NR_178998.1	Bee bread
Lactobacillus melliventris	NR_126252.1	*Apis mellifera* honey stomach
Lactobacillus huangpiensis	NR_179358.1	*Apis mellifera* gut
Lactobacillus laiwuensis	NR_179376.1	*Apis mellifera* gut
Lactobacillus kimbladii	NR_126250.1	*Apis mellifera* honey stomach
Amylolactobacillus amylopilus	NR_113816.1	Type strain of *Amylolactobacillus amylopilus*
	Streptococcacea tree	
Streptococcaceae bacterium	CP113940.1	*Melipona seminigra* gut
Streptococcaceae strain MM128	Non-published	*Melipona mondury* gut
Floricoccus tropicus	NR_159226.1	Flowers of durian tree and *Hibiscus*
Floricoccus penangensis	NR_159225.1	Flowers of durian tree and *Hibiscus*
Streptococcaceae bacterium	CP113924.1	*Scaptotrigona polystica* gut
Lactococcus piscium	NR_043739.1	Broiler carcasses
Lactococcus plantarum	NR_044358.1	Activated sludge foam
Lactococcus lactis	NR_113958.1	Type strain *Lactococcus lactis subsp. Hordniae*

Lactococcus taiwanensis	NR_114327.1	Pobuzihi, traditional fermented food in Taiwan
Streptococcus agalactiae	NR_113262.1	Type strain of Streptococcus agalactiae
Streptococcus tangierensis	NR_134818.1	Raw camel milk
Streptococcus cameli	NR_134817.1	Raw camel milk
Streptococcus thoraltensis	NR_026368.1	Genital tract of sows
Streptococcus hyovaginalis	NR_044912.1	Genital tract of sows
Lactiplantibacillus plantarum	NR_113338.1	Type strain of *Lactiplantibacillus plantarum*
Bifidobacteriaceae tree		
Bifidobacterium minimum	NR_044692.2	Sewage
Bifidobacterium indicum	NR_043439.1	Hindgut of honeybee
Bifidobacterium callitrichos	NR_113172.1	Faeces of common marmoset (*Callithrix jacchus*)
Bifidobacterium rousetti	NR_164634.1	Faeces of Egyptian fruit bat
Bifidobacterium asteroids	NR_044154.1	*Apis mellifera* honey stomach
Bifidobacterium bombi	NR_104872.1	Digestive tract of *Bombus lucorum*
Bifidobacterium bohemicum	NR_108439.1	Bumblebee digestive tract
Bifidobacterium sp. ESL0798	CP113914.1	*Scaptotrigona polysticta* gut
Bifidobacterium sp. ESL0728	CP113925.1	*Melipona fuliginosa* gut
Bifidobacterium sp. ESL0704	CP113929.1	*Scaptotrigona* sp. gut
Bifidobacterium sp. ESL0790	CP113915.1	*Melipona fuliginosa* gut
Bifidobacterium sp. ESL0690	CP113939.1	*Melipona lateralis* gut
Bifidobacterium sp. ESL0682	CP113942.1	*Melipona fuliginosa* gut
Bifidobacterium commune	NR_136422.1	Bumblebee gut
Bifidobacteium sp. ESL0769	CP113918.1	*Melipona fuliginosa* gut
Bifidobacterium sp. ESL0764	CP113919.1	*Scaptotrigona polysticta* gut
Bifidobacterium sp. ESL0732	CP113920.1	*Scaptotrigona polysticta* gut
Bombiscardovia coagulans	NR_116179.1	Bumblebee digestive tract
Acetobacteraceae tree		
Asaia atilbis	NR_122089.1	Japanese flowers
Asaia krungthepenses	NR_024810.1	*Heliconia* sp. flower
Nguyenibacter vanlangensis	NR_125459.1	Rhizosphere of Asian rice
Commensalibacter melissae	CP046393.1	Honeybee gut
Commensalibacter intestini	NR_116307.1	*Drosophila melanogaster* gut
Acetobacter tropicalis	NR_036881.1	Type strain of *Acetobacter tropicalis*
Neokomagataea tanensis	NR_112959.1	Candle bush flower
Neokomagataea anthophila	MZ165367.1	Flower
Oecophyllibacter saccharovorans	NR_173550.1	Weaver ant *Oecophylla smaragdina*
Saccharibacter floricola	NR_024819.1	Pollen
Bombella mellum	NR_181315.1	Honeycomb of *Apis mellifera*
Bombella apis	NR_157653.1	Midgut of honeybee
Bombella favorum	NR_181314.1	Honeycomb of *Apis mellifera*
Bombella intestini	NR_178684.1	Bumblebee crop
Bombella sp. strain ESL0368	CP046394.1	Honeybee gut
Granulibacter bethesdensis	NR_074276.1	Type strain of *Granulibacter bethesdensis*
