# Supplementary material for: Gut microbiota of Brazilian Melipona stingless bees: Dominant members and their localization in different gut regions
Source: PLoS One. 2026 May 7;21(5):e0326546. doi: 10.1371/journal.pone.0326546 (PMC13152157; doi:10.1371/journal.pone.0326546)
Supplement: S5 Table — (PDF) [file pone.0326546.s005.pdf]

S5 Table. Results of Kruskal–Wallis and Dunn's post hoc tests comparing alpha diversity (Shannon index) across gut regions of *Melipona quadrifasciata*. The global Kruskal–Wallis test indicated significant differences among gut regions ($\chi^2 = 14.9$, df = 3, $p = 0.0019$). Pairwise comparisons were performed using Dunn's test with Benjamini–Hochberg correction for multiple testing.

Comparison	Z_statistic	p_uncorrected	p_BH_adjusted	Significant
crop - ileum	-2.432	0.00751	0.0225	Yes
crop - rectum	1.391	0.0821	0.0985	No
ileum - rectum	3.758	8.55E-05	0.000513	Yes
crop - ventriculus	-0.843	0.2	0.2	No
ileum - ventriculus	1.589	0.0561	0.0841	No
rectum - ventriculus	-2.212	0.0135	0.027	Yes
