# Supplementary material for: Gut microbiota of Brazilian Melipona stingless bees: Dominant members and their localization in different gut regions
Source: PLoS One. 2026 May 7;21(5):e0326546. doi: 10.1371/journal.pone.0326546 (PMC13152157; doi:10.1371/journal.pone.0326546)
Supplement: S6 Table — (PDF) [file pone.0326546.s006.pdf]

S6 Table. Results of Kruskal–Wallis and Dunn's post hoc tests comparing alpha diversity (richness) across gut regions of *Melipona quadrifasciata*. The global Kruskal–Wallis test indicated no significant differences among gut regions ($\chi^2 = 5.061$, df = 3, $p = 0.167$). Pairwise comparisons were performed using Dunn's test with Benjamini–Hochberg correction for multiple testing.

Comparison	Z_statistic	p_uncorrected	p_BH_adjusted	Significant
crop - ileum	-2.06	0.0197	0.118	No
crop - rectum	-0.227	0.41	0.41	No
ileum - rectum	1.778	0.0377	0.113	No
crop - ventriculus	-0.638	0.262	0.393	No
ileum - ventriculus	1.422	0.0775	0.155	No
rectum - ventriculus	-0.394	0.347	0.416	No
