# Supplementary material for: Gut microbiota of Brazilian Melipona stingless bees: Dominant members and their localization in different gut regions
Source: PLoS One. 2026 May 7;21(5):e0326546. doi: 10.1371/journal.pone.0326546 (PMC13152157; doi:10.1371/journal.pone.0326546)
Supplement: S1 Fig — (PDF) [file pone.0326546.s007.pdf]

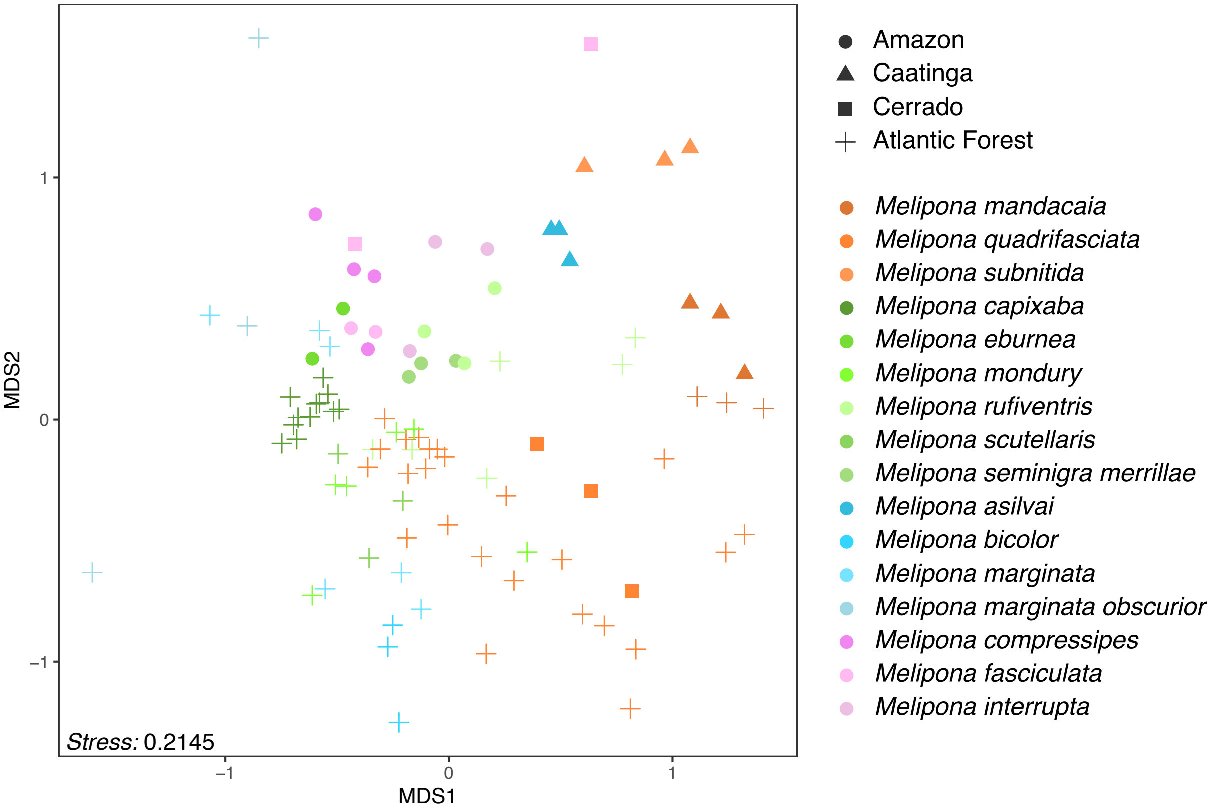

S1 Figure. NMDS plot based on ASV relative abundance using a Bray-Curtis dissimilarity matrix, illustrating bacterial community composition across different *Melipona* species and biomes. Colors represent bee species, with color groupings indicating *Melipona* subgenera: orange – *Melipona*, green – *Michmelia*, blue – *Eomelipona*, and pink – *Melikerria*. Point shapes denote the biome of origin.
