# Supplementary material for: Gut microbiota of Brazilian Melipona stingless bees: Dominant members and their localization in different gut regions
Source: PLoS One. 2026 May 7;21(5):e0326546. doi: 10.1371/journal.pone.0326546 (PMC13152157; doi:10.1371/journal.pone.0326546)
Supplement: S4 Fig — (PDF) [file pone.0326546.s010.pdf]

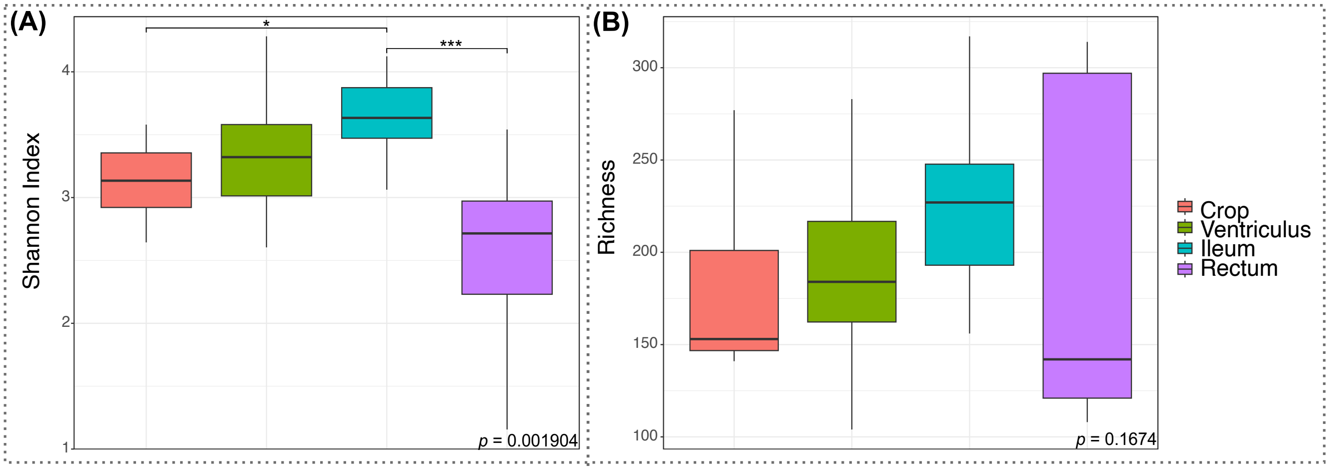

S4 Figure. Bacterial alpha diversity of the gut regions of *M. quadrifasciata*. The alpha diversity was expressed using the Shannon and richness indexes. A Kruskal-Wallis test ($p < 0.05$) was conducted, followed by a post-hoc pairwise Dunn test to compare each gut part, showing only the significant results.
