# Supplementary material for: Gut microbiota of Brazilian Melipona stingless bees: Dominant members and their localization in different gut regions
Source: PLoS One. 2026 May 7;21(5):e0326546. doi: 10.1371/journal.pone.0326546 (PMC13152157; doi:10.1371/journal.pone.0326546)
Supplement: S5 Fig — (PDF) [file pone.0326546.s011.pdf]

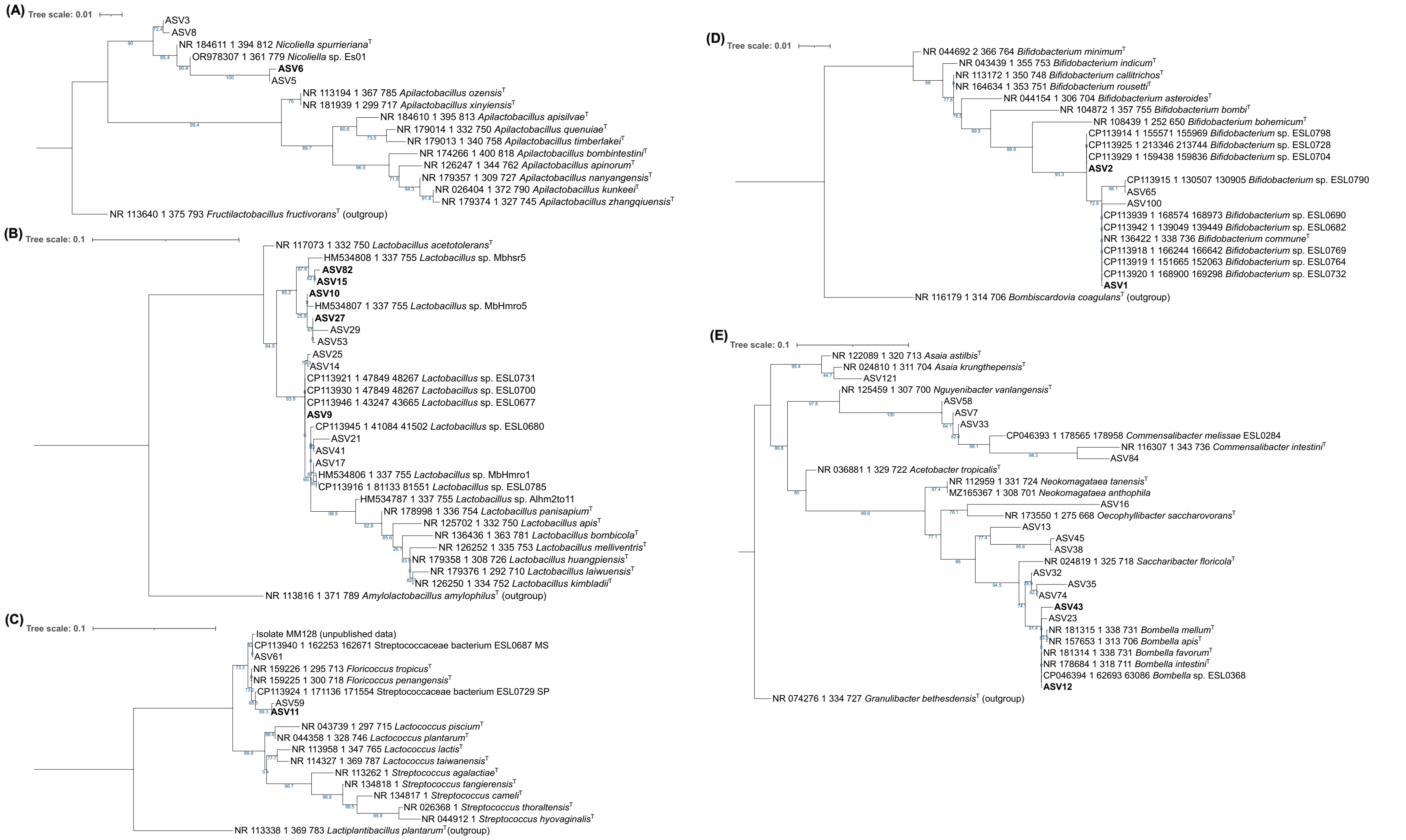

S5 Figure. Phylogenetic trees of the most abundant ASVs (including the 11 core ASVs) found in *Melipona* bee populations. Bootstrap values are shown in blue letters. The 11 core ASVs are written in bold characters. ^T Type strain. Trees are shown for the most abundant and core ASVs of A) *Apilactobacillus*, B) *Lactobacillus*, C) Streptococcaceae, D) Bifidobacteriaceae, and E) Acetobacteraceae. The phylogenetic trees were rooted according to the outgroups: (A) *Fructilactobacillus fructivorans*, (B) *Amylolactobacillus amylophilus,* (C) *Lactiplantibacillus plantarum*, (D) *Bombiscardovia coagulans*, (E) *Granulibacter bethesdensis*.
