# Supplementary material for: Gut microbiota of Brazilian Melipona stingless bees: Dominant members and their localization in different gut regions
Source: PLoS One. 2026 May 7;21(5):e0326546. doi: 10.1371/journal.pone.0326546 (PMC13152157; doi:10.1371/journal.pone.0326546)
Supplement: S2 Fig — Each sample represents a pool of 5 bees per box per site of study. (PDF) [file pone.0326546.s008.pdf]

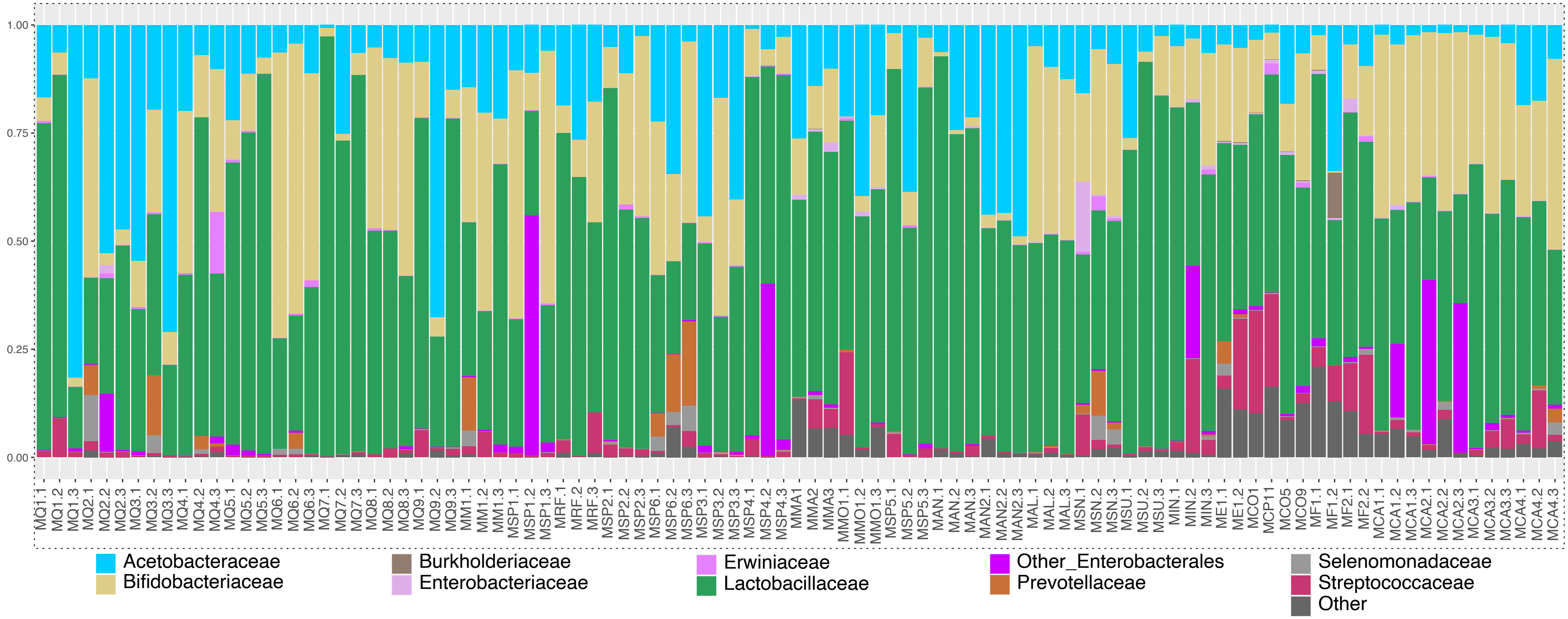

S2 Figure. Most abundant families in *Melipona* spp. gut microbiota. Each sample represents a pool of 5 bees per box per site of study. ASVs are ordered and colored at the family level, with low abundant ASVs grouped as 'Other'.
